# Efficacy Comparison of Different Acupuncture Treatments for Primary Insomnia: A Bayesian Analysis

**DOI:** 10.1155/2019/8961748

**Published:** 2019-09-03

**Authors:** Huachong Xu, Yucong Shi, Yike Xiao, Pei Liu, Sizhi Wu, Peng Pang, Li Deng, Xiaoyin Chen

**Affiliations:** School of Traditional Chinese Medicine, Jinan University, Guangzhou 510632, China

## Abstract

**Background:**

Acupuncture treatments are used frequently in the treatment of primary insomnia considering its less side effect. However, most treatment choices are made just based on personal experience among different forms of acupuncture. This study compared the effectiveness of different forms of acupuncture for primary insomnia by using network meta-analysis.

**Methods:**

All randomized controlled trials (RCTs) of acupuncture treatments for primary insomnia were searched in seven databases from the date of database inception to January 6, 2019, including PubMed, Web of Science, Embase, Cochrane Library, Wanfang database, China National Knowledge Infrastructure (CNKI) database, and VIP Chinese Science and Technique Journals (CQVIP) database. After screening, the effectiveness rate was extracted from the included RCTs as primary outcomes. The network meta-analysis was performed by Review Manager 5.3, Stata13.0, and GeMTC 0.14.3.

**Results:**

Forty-two studies were included, which contained 3304 participants among 6 interventions. Based on the ranking probability and compared to western medicine, scalp acupuncture (OR = 8.12, 95% CI (4.07,16.81)) is considered to be the most effective method, followed by electroacupuncture (OR = 6.29, 95% CI (3.36, 12.67)), electroacupuncture combined scalp acupuncture (OR = 5.20, 95% CI (2.43,11.28)), warm acupuncture (OR = 3.79, 95% CI (1.85,8.16)), and conventional acupuncture (OR = 2.86, 95% CI (2.05,3.95)). There was no significant difference between the results of direct and indirect comparisons.

**Conclusions:**

The finding indicated that five acupuncture methods may be all effective in the treatment of primary insomnia, and scalp acupuncture seems to be the best treatment. However, the overall quality of the included trials could only be ranked as medium to low quality, and higher quality RCTs are warranted for sufficient evidence.

## 1. Introduction

Primary insomnia is one of the most common clinical diseases in the world, which is defined as sleeplessness without a medical, psychiatric, or environmental cause [1, 2]. Approximately 30% of the world's adults have been plagued by sleep disorders, and 6 to 10 percent can be diagnosed as insomnia [3, 4]. Long-term insomnia mostly causes physical and psychological damage, which increases the risk of other diseases [5, 6], reduces the quality of life [7], and ultimately causes psychological [8, 9] and economic burden [10, 11]. As a public health problem, insomnia has not received enough recognition and has not been effectively resolved.

Currently, treatments for primary insomnia include medications and nondrugs. Medications, including benzodiazepines, melatonin, and other sedatives, can alleviate some of the symptoms but often cause adverse reactions such as excessive sedation, tolerance, addiction, and neurological toxicity [12, 13]. Cognitive-behavioral therapy (CBT) is recommended as an effective nondrug therapy [14], but it is difficult to popularize due to its rare resources and expensive costs [15, 16].

In China, acupuncture is considered as an effective alternative treatment for insomnia because of its low side effects and easy availability. As a safe and effective natural therapy, acupuncture therapy has been added to the latest version of Guidelines for the Diagnosis and Treatment of Insomnia in China [17] by the China Sleep Research Association. Meanwhile, many clinical randomized trials [18–21] and systematic analysis [22–26] have shown that a variety of acupuncture treatments are significantly better than drug therapy in primary insomnia. There are many types of acupuncture treatment, including conventional acupuncture [21], warm acupuncture [27], electroacupuncture [28], scalp acupuncture [29], combined acupuncture [30], and so on. However, these previous studies have only demonstrated the effectiveness of single acupuncture treatment against their own control intervention for primary insomnia. Meanwhile, these previous systematic reviews [22–26] have only considered all the acupuncture treatments as a whole to study its effectiveness. Clinically, the choice of different acupuncture therapies is often based on the doctor's personal experience. Lack of authoritative selection guidelines may often lead to an unsatisfactory effect and longer course of treatment for acupuncture therapies. Therefore, the comparison of the efficacy is still lacking and necessary among various acupuncture therapies.

With more new research evidence being included, a network meta-analysis was performed to critically evaluate the current evidence from RCTs involving conventional acupuncture, scalp acupuncture, electroacupuncture, warm acupuncture, electroacupuncture combined scalp acupuncture, and western medicine. This study was committed to comparing the effectiveness of different acupuncture treatments for primary insomnia and finding a better acupuncture treatment selection guideline for clinicians and patients.

## 2. Methods

### 2.1. Search Strategy

**Figure 1:**
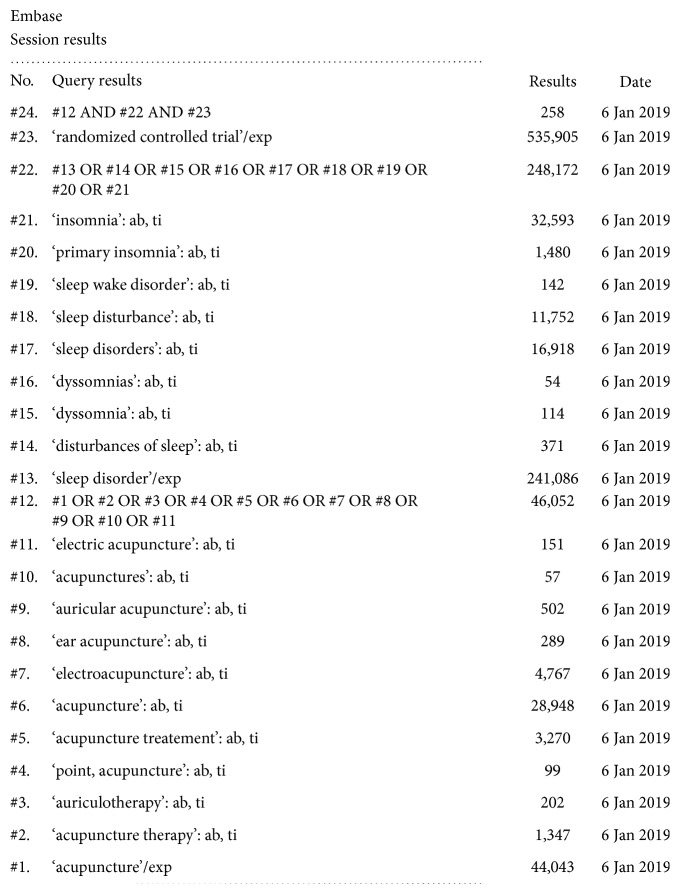
The search strategy for Embase.

Seven electronic databases including PubMed, Web of Science, Embase, Cochrane Library, Wanfang database, China National Knowledge Infrastructure (CNKI) database, and VIP Chinese Science and Technique Journals (CQVIP) database were systematically searched from the date of database inception to January 6, 2019, to identify eligible RCTs. The languages of the trials were restricted to English or Chinese. The following keywords were used in search strategies: (“Acupuncture” OR “Acupuncture Therapy” OR “Scalp Acupuncture” OR “Electroacupuncture” OR “Warm Acupuncture”) AND (“Insomnia” OR “Sleep Initiation and Maintenance Disorders”) AND (“Randomized Controlled Trial” OR “Randomized”). The search strategy for each database was based on its own unique characteristics. The search strategy for Embase is shown in Figure 1, and similar search terms were adopted for the other databases.

### 2.2. Inclusion Criteria

#### 2.2.1. Types of Trials

The included study must be a clinical randomized controlled trial published in the journal, with languages limited to Chinese and English.

#### 2.2.2. Participants

Participants must be between 18 and 75 years of age, regardless of gender, disease duration, and severity. Patients with primary insomnia should be diagnosed with at least one of the international or domestic authorized diagnostic criteria, such as International Classification of Disease Tenth Revision (ICD-10), the Chinese Classification of Mental Disorders Third Revision (CCMD-3), and the Chinese Classification of Mental Disorders Second Edition-Revision (CCMD-2-R).

#### 2.2.3. Interventions and Comparison

The interventions for the experimental group must be conventional acupuncture, electroacupuncture, warm acupuncture, scalp acupuncture, or a combination treatment of these methods; additionally, the interventions for the control group were western medicine or other acupuncture treatment different from the experimental group. Western medicine must be drugs recommended in international authorized clinical guidelines.

#### 2.2.4. Outcomes

Effectiveness rate was the primary outcome. Effectiveness rate is reported by measuring insomnia symptom improvement according to standards of five different versions. Effectiveness includes those who have recovered and have a significant improvement over their original symptoms. Five different version standards [23] include Guiding Principles for Clinical Study of New Chinese Medicines, Standards for Diagnosis and Curative Effect of Chinese Medical Symptom, Sleep Efficiency Calculation published by WHO, Guiding Principles for Clinical Study of New Chinese Medicines combined with PSQI reducing rate, and PSQI reducing rate.

### 2.3. Exclusion Criteria

Exclusion criteria include (1) duplicate studies; (2) insomnia being identified with a clear cause such as medical, psychiatric, or environmental cause; (3) studies without clear outcomes for effectiveness rate; (4) trials that failed to offer accurate data for extraction; and (5) trials with low readability.

### 2.4. Study Selection and Data Extraction

Study selection and data extraction were conducted with Endnote X9 and Excel 2018. Firstly, study titles and abstracts were screened by two independent reviewers after removing duplicate studies in EndNote. Secondly, they would read the full text of relevant studies after title-abstract screen, according to inclusion and exclusion criteria. If any disagreement arises, the final decision would be made by the third reviewer. Finally, included studies would be coded and extract the following data: study characteristics (author and year of publication); participant characteristics (diagnose criteria, age, disease course, and cases of each group); intervention information (measures of intervention and control, treatment duration, follow-up period, and adverse events); and outcome (definition used in the study).

### 2.5. Study Quality Evaluation

According to the Cochrane risk of bias assessment tool [31], the quality evaluation of included RCTs was conducted by two reviewers independently using Review Manager 5.3. The following aspects were evaluated: (1) random sequence generation; (2) allocation concealment; (3) blinding of participants and personnel; (4) blinding of outcome assessment; (5) incomplete outcome data; (6) selective reporting; and (7) other bias. Any disagreements would be analyzed by the third reviewer.

### 2.6. Statistical Analysis

Odds ratio (OR) was adopted for dichotomous outcomes (effectiveness rate) in all studies. The confidence interval (CI) was set at 95%; *P* < 0.05 was regarded as statistically significant. Statistical evaluation of inconsistency and production of network graphs were conducted using the network and network graphs packages in Stata 13.0. The results of the inconsistency test would be used to decide whether to adopt a consistency model.

Then, Bayesian analysis was performed using GeMTC 0.14.3 (Generate Mixed Treatment Comparisons) with the MCMC (Markov Chain Monte Carlo) method, and it estimated the posterior probability according to the prior probability. Estimations and inferences would be conducted when the MCMC has reached a stable convergence state. The parameters of GeMTC are set as follows: the initial value is set to 2.5; the number of simulation iterations is set to 50,000; 20,000 adjustment iterations are performed first to eliminate the influence of the initial value; and the step size (sparse interval) is set to 10 when the number of chains is 4. The potential scale reduced factor (PSRF) reflects the convergence of the model. When the PSRF is close to 1 (means the convergence is good), the consistency of the homogeneity model would be considered reliable enough for follow-up analysis. Finally, the figure of ranking probability was generated for all interventions and the node-splitting method was adopted to evaluate local inconsistency.

## 3. Results

### 3.1. Literature Search Results

**Figure 2:**
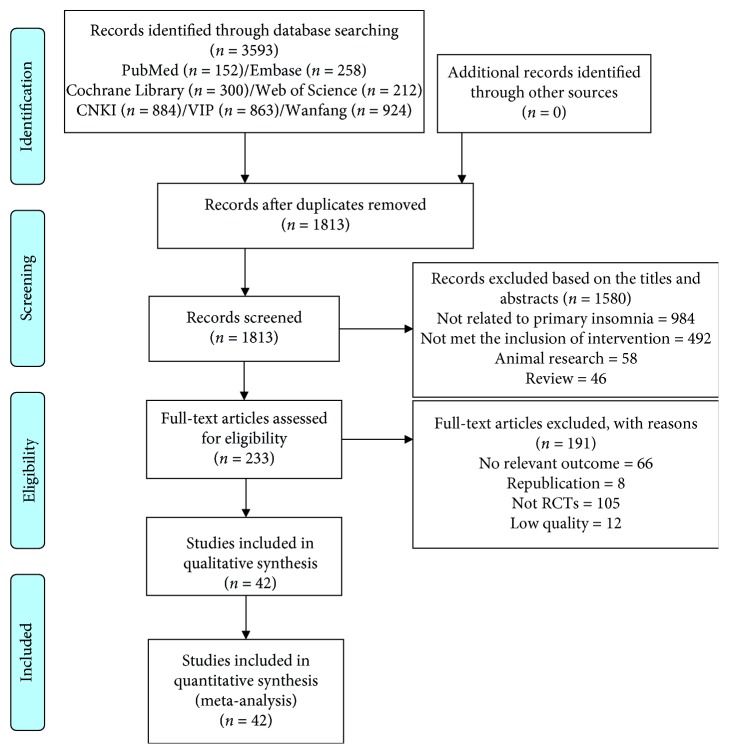
Study selection process.

A total of 3593 records were identified through database searching. After duplicates removed and titles-abstracts screened, 233 potentially eligible studies were retrieved in full text. Based on full-text screening, 191 papers were excluded with the following reasons: have no relevant outcome; republication; no RCTs; or low-quality studies. Finally, 42 RCTs were included in the Bayesian analysis. 3 of them are in English, and the other 39 are in Chinese including 30 articles from the catalogue of statistical sources of Chinese scientific papers (considered to be better journals in China). The specific literature search and screening process are presented in the PRISMA 2009 Flow Diagram (as shown in Figure 2).

### 3.2. Study Characteristics

Forty-two included RCTs were done between 2004 and 2018 containing a total of 3304 participants. There were 1686 patients in the intervention group and 1618 in the control group. The interventions of 42 trials included conventional acupuncture, electroacupuncture, scalp acupuncture, warm acupuncture, and electroscalp acupuncture, of which 18 were treated with conventional acupuncture, 6 with electroacupuncture, 7 with scalp acupuncture, 5 with warm acupuncture, and 6 with electroscalp acupuncture. In addition, the control group of 34 trials was treated with western drugs, and the remaining 8 trials were treated with conventional acupuncture. The duration of treatment for these studies ranged from 10 days to 8 weeks. The drugs included estazolam (24 trials), clonazepam (2 trials), zopiclone (2 trials), diazepam (3 trials), alprazolam (1 trial), zolpidem (1 trial), and nitrazepam (1 trial). All included trials have clear diagnostic criteria and outcome criteria. Characteristics of included studies are shown in Table 1.

### 3.3. Adverse Event and Follow-Up

Adverse events were mentioned in 7 trials. Two trials reported minor bleeding in the acupuncture group, but the spirit was good; the western medicine group had poor mentality during the day [19, 43]. One trial detailed the adverse events: 1 patient in the acupuncture treatment group felt nausea without vomiting, 2 patients had migraine headache, and the symptoms were relieved after rest; in the Western medicine group, there were 3 patients with obvious mouth pain, 5 patients with dizziness, nausea, and appetite, and 3 patients with decreased disease [44]. The other trial found that 2 patients in the acupuncture treatment group had fainting due to long retention, hypoglycemia, and symptom relief after rest [39]. Another trial found that 4 patients in the acupuncture group felt pain, which could be resolved by acupuncture adjustment and verbal communication; 10 patients in the control group had a wake-up headache; and 7 patients developed dizziness [53]. One of the trials clearly indicated that the acupuncture treatment group was safer than the drug control group, while the other trial concluded that both interventions were equally safe [27, 54]. No patient was reported to withdraw from the study due to adverse events.

Follow-up was adopted to only 5 trials [19, 47, 50, 51, 56] for lasting effect ranging between 2 weeks and 3 months. Two studies concluded that the long-term efficacy of the acupuncture group was better than that of the drug group. Two studies suggested that the recurrence rate of the treatment group was lower than that of the control group. The remaining study showed no significant difference of efficacy between the treatment group and the control group after 3 months of follow-up.

### 3.4. Risk of Bias for Research Quality Evaluation

**Figure 3:**
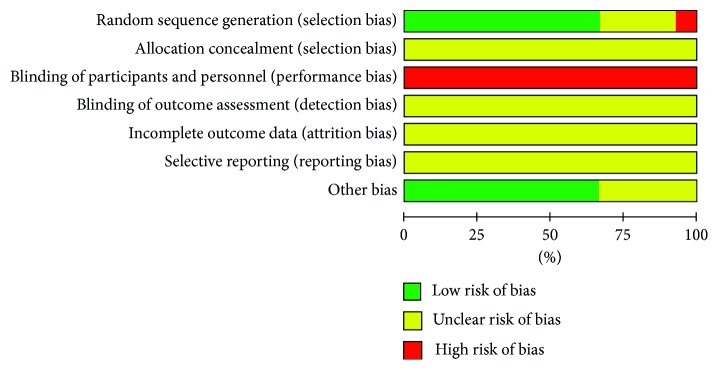
Risk of bias graph: review authors' judgements about each risk of bias item presented as percentages across all included studies.

**Figure 4:**
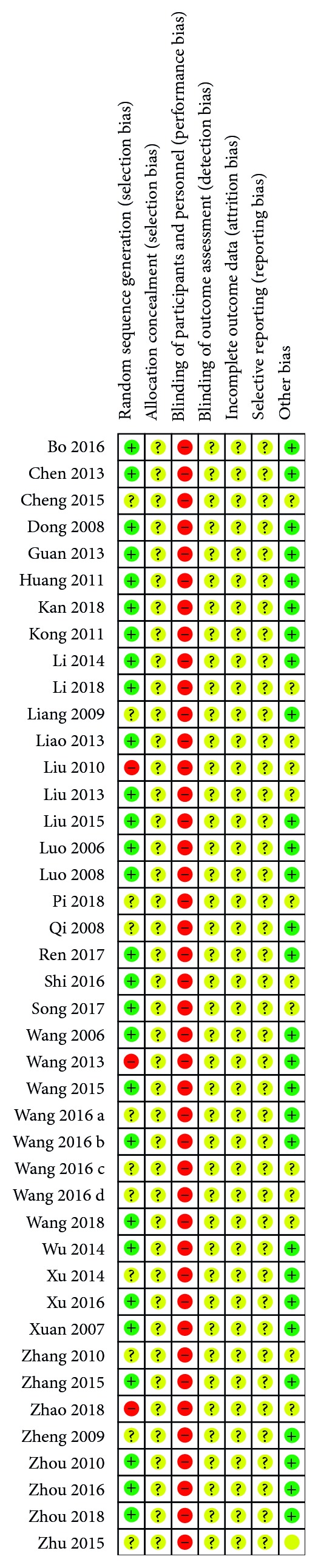
Risk of bias summary: review authors' judgements about each risk of bias item for each included study (reproduced from Peng Pang et al. [66] (under the Creative Commons Attribution License/Public Domain)).

The assessment results showed a medium quality for all the included literatures. All studies referred to randomization, but only 28 studies described detailed and reliable random grouping methods, 23 of which used random number tables and 5 used computer software. Both concealment of allocation and blinding of outcome were not mentioned in all studies. Due to the special nature of acupuncture treatment, blinding was not used in all studies. Therefore, there is a high risk of performance bias in literature quality assessment. The detailed evaluation is shown in Figures 3 and 4.

### 3.5. Network Map for Interventions

**Figure 5:**
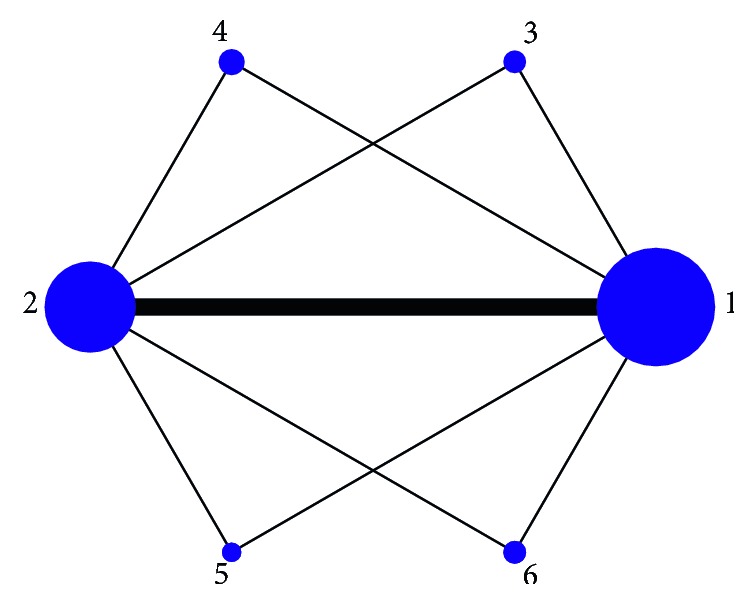
Network meta-analysis of eligible comparisons for efficacy. Note: 1, western medicine; 2, conventional acupuncture; 3, electroacupuncture; 4, scalp acupuncture; 5, warm acupuncture; 6, electroacupuncture combined scalp acupuncture.

Network map among 6 interventions was made by Stata 13.0 (as shown in Figure 5). The size of the points in the graph is proportional to the weight of the sample number of interventions, and the thickness of the lines in the figure is also proportional to the correlation between the two interventions. The figure shows that the sample of conventional acupuncture and western medicine ranked in the top two in this study. The other four interventions are directly compared with conventional acupuncture and western drugs, but there are no direct comparisons between any two of them. Thus, the network meta-analysis was performed to combine direct comparison with indirect comparison.

### 3.6. Results of Network Meta-Analysis

According to the result of the inconsistency test (*P*=0.416 > 0.05), there is no significant heterogeneity in the data. The local inconsistency test was shown in loop inconsistency map: all the 95% CIs include zero, and all the IF are close to zero. Hence, a consistency model was selected for the network meta-analysis, and the results are shown in Table 2.

Firstly, compared with western medicine, the following interventions can significantly improve the effectiveness of primary insomnia treatment: conventional acupuncture (OR = 2.86, 95% CI (2.05, 3.95), *P* < 0.05), electroacupuncture (OR = 6.29, 95% CI (3.36, 12.67), *P* < 0.05), scalp acupuncture (OR = 8.12, 95% CI (4.07,16.81), *P* < 0.05), warm acupuncture (OR = 3.79, 95% CI (1.85, 8.16), *P* < 0.05), and scalp acupuncture plus electroacupuncture (OR = 5.20, 95% CI (2.43, 11.28), *P* < 0.05). Secondly, compared with conventional acupuncture, the following methods showed significant improvement: electroacupuncture (OR = 2.18, 95% CI (1.10, 4.68), *P* < 0.05) and scalp acupuncture (OR = 2.86, 95% CI (1.48, 5.72), *P* < 0.05). The remaining paired comparisons showed no significant differences in effectiveness rate of treatment.

### 3.7. Comparison of the Effectiveness of Different Interventions

**Figure 6:**
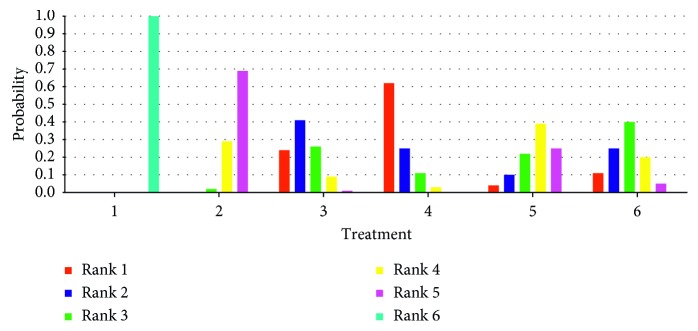
The rank probability of efficacy for included interventions. *Note*. 1, western medicine; 2, conventional acupuncture; 3, electroacupuncture; 4, scalp acupuncture; 5, warm acupuncture; 6, electroacupuncture combined scalp acupuncture.

The figure of ranking probability was generated based on the MCMC theory for probability evaluation (Figure 6). In the six treatments for primary insomnia included in this study, scalp acupuncture is considered to be the most effective method, followed by electroacupuncture, electroacupuncture combined scalp acupuncture, warm acupuncture, conventional acupuncture, and finally western medicine.

According to the Gelman Rubin-Brooks diagnostic method, the convergence diagnostic plot was drawn and it showed that the median value of the reduction factor and 97.5% tend to be stable after 25,000 iterations, and then the Bayesian model was calculated to 25,000 iterations. The parameter PSRF moves close to 1, indicating satisfactory convergence (Table 3). Finally, the local inconsistency was performed using the node-splitting method. The *P* values of all the comparison groups were greater than 0.05, indicating that the direct comparisons were consistent with the indirect comparisons (Table 4).

### 3.8. Publication Bias

**Figure 7:**
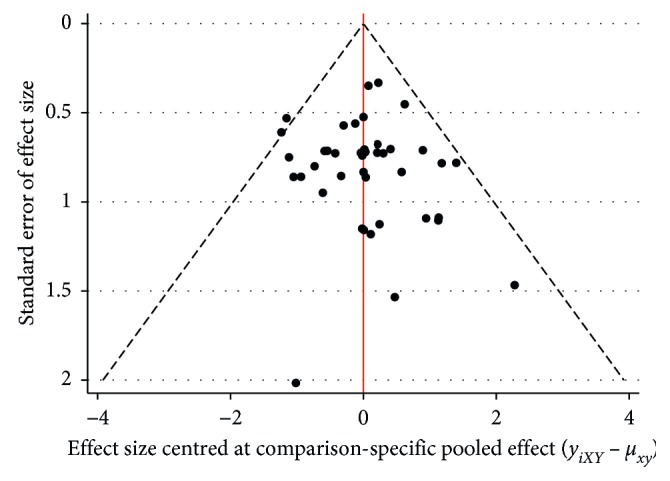
Comparison-adjusted funnel plot for the network meta-analysis.

**Figure 8:**
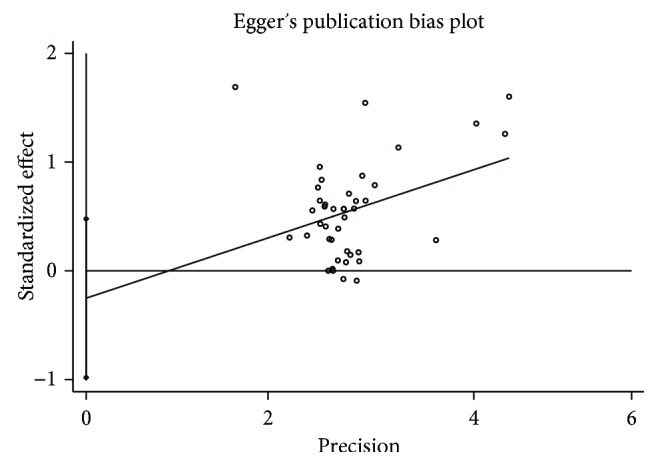
Egger's publication bias plot (*P*=0.490 > 0.05).

According to the funnel plot (Figure 7), we found no obvious publication bias. Furthermore, we conducted verification by Egger's test (Figure 8) and found no publication bias (*P*=0.490 > 0.05).

## 4. Discussion

Repeated insomnia may have many, yet largely unknown, repercussions for health and well-being. Most clinical studies and experimental studies have shown that acupuncture is effective for primary insomnia. Thus, this study combines the results of the previous studies with network meta-analysis to compare the efficacy of different acupuncture methods in the treatment of primary insomnia and finally rank them according to the efficacy.

As of now, this study is the first network meta-analysis of primary insomnia. Different from previous systematic analysis [22–26], it has incorporated the direct comparison and indirect comparison of various acupuncture therapies into the study. The internal comparison of acupuncture therapy is more helpful in exploring the specific role and connotation of them. Before the study, we found that a previous protocol [67] on the network meta-analysis of acupuncture for primary insomnia has been published. And, in accordance with the review protocol, we made some improvements based on actual conditions. In the inclusion criteria, in order to improve the homogeneity and feasibility of the research results, the control drug was designated as western medicine and does not include traditional Chinese medicine or proprietary Chinese medicine with unclear mechanism of action. In the choice of software, we use GeMTC instead of Stata for the final simulation iteration, of which the iterative conditions are stricter and more stable, so the results obtained have higher feasibility. Actually, results of the PSRF and the test of local inconsistency had proved that an appropriate model has been successfully established.

Since ancient times, acupuncture has been used to treat primary insomnia in China. Chinese medicine believes that the occurrence of insomnia is closely related to mood, constitution, disease, and environment and ultimately blamed to the imbalance between yin and yang. Acupuncture is believed to balance the body and restore its physiological function by inserting thin needles at specific acupoints [68]. Moreover, the modern biological mechanism of acupuncture treatment of primary insomnia is not fully revealed. Studies have shown that acupuncture can regulate some insomnia by regulating some neurotransmitters [23, 69], such as norepinephrine, serotonin, dopamine, acetylcholine, and *γ*-aminobutyric acid [70], and reducing glutamate levels [71]. Acupuncture may also increase melatonin, which involves sleep-wake adjustment [72, 73]. Acupuncture can improve sleep quality by enhancing the blood supply to brain tissue, the elasticity of blood vessels, and the excitability of related sites on the cerebral cortex [23]. Consistent with the above existing theory, scalp acupuncture is considered to be the best treatment for primary insomnia in this study. Experimental studies have found that sleep and wakefulness regulation is one of the basic functions of the brain, and there are specific sleep-inducing regions in the brain [7]. As a novel treatment using traditional acupuncture and modern medical cerebral cortical positioning theory, scalp acupuncture is a method of acupuncture treatment of certain areas of the scalp to treat primary insomnia. Additionally, although both the scalp acupuncture and the electroacupuncture have a good therapeutic effect, the combined treatment of them seems to be less effective, which may be caused by the small number of studies in the combination therapy. Lastly, we found that all acupuncture treatments are better than medications while only two of them have significant difference with conventional acupuncture. At the same time, there is no significant difference between any two of the other four acupuncture treatment besides conventional acupuncture, which may need more clinical trials to be carried out.

Our study still has some limitations. Firstly, the overall quality of the included trials could only be ranked as medium to low quality, which usually affects the strength of the evidence to some extent. Higher quality clinical RCTs should be performed, with reference to authoritative criteria for random methods, allocation concealment, blinding methods, and so on. Secondly, acupoint selection between acupunctures of different therapies or between the same therapy may be inconsistent and empirical, and the techniques of acupuncturists may differ, both of which may increase the heterogeneity of the study. Future clinical studies can focus on studying the effects of specific acupoints on the treatment of insomnia, but this may be contradictory with the principle of acupuncture treatment based on syndrome differentiation. Finally, most of the included studies tend to have short treatment duration and lack of follow-up, which is still not enough to indicate the long-term effect of acupuncture. On the whole, higher quality RCTs are warranted.

## 5. Conclusion

This study indicated that the included five acupuncture methods may be effective and safe in improving the condition of patients with primary insomnia, and scalp acupuncture seems to be a better treatment for primary insomnia. However, the overall quality of the included trials could only be ranked as medium to low quality, and higher quality RCTs are warranted for sufficient evidence.

## Figures and Tables

**Table 1:** Characteristics of included studies.

Studies (year)	Diagnostic criteria	Mean age (years) (T/C)	Course of disease (T/C) (y, years; m, months; d, days)	Cases (T/C)	Intervention			Treatment time	Efficacy criteria	Follow-up and AEs
					Treatment	Control				
Bo et al. (2016) [18]	ICSD-2	(43.25 ± 9.56)/(47.21 ± 8.31)	(3.15 ± 1.24)/(4.10 ± 1.06) y	40/40	W-ACU		Estazolam	8 weeks	V5	—
Chen et al. (2013) [19]	CCMD-3	(33 ± 12)/(35 ± 12)	(25.4 ± 26.6)/(33.4 ± 52.3) m	107/104	C-ACU		Estazolam	2 weeks	V4	2 weeks, AEs
Cheng et al. (2015) [20]	V1	52.9/54.1	(2.7 ± 1.6)/(2.9 ± 1.9) y	63/61	S-ACU		Estazolam	20 days	V1	—
Dong et al. (2008) [32]	CCMD-2R	(57.4 ± 9.1)/(58.4 ± 8.6)	(2.7 ± 2.1)/(2.8 ± 2.65) y	36/35	S-ACU		C-ACU	30 days	V1	—
Guan et al. (2013) [21]	ICD-10	(14 ∼ 65)/(15 ∼ 66)	(1 ∼ 10)/(1 ∼ 10) y	40/40	C-ACU		Diazepam + oryzanol + VB1	20 days	V1	—
Huang et al. (2011) [33]	CCMD-3	40/38	(17/19) m	30/30	S-ACU		Estazolam	20 days	V1	—
Kan et al. (2018) [27]	CCMD-3	(49.74 ± 8.64)/(48.63 ± 8.44)	(166.42 ± 27.48)/(171.38 ± 36.42) d	35/35	W-ACU		Estazolam	6 weeks	V1	AEs
Kong et al. (2011) [34]	V1	(30 ∼ 62)/(28 ∼ 64)	(2m ∼ 6 y)/(2m ∼ 7 y)	54/52	C-ACU		Diazepam	20 days	V1	—
Li et al. (2014) [28]	CCMD-3	53.5/54.5	(4.8/6.2) y	30/30	E-ACU		Estazolam	20 days	V1	—
Li et al. (2018) [29]	CCMD-3	(43.07 ± 7.51)/(43.33 ± 7.08)	(5.64 ± 2.24)/(5.96 ± 1.86) m	30/30	S-ACU		C-ACU	4 weeks	V1	—
Liang et al. (2009) [35]	V2	42.3/40.8	(5.6/5.1) y	40/30	E + S-ACU		C-ACU	10 days	V1	—
Liao et al. (2013) [36]	CCMD-2-R	(45.23 ± 14.10)/(43.10 ± 12.16)	(14.27 ± 7.78)/(16.47 ± 7.49) d	30/30	E-ACU		Estazolam	20 days	V4	—
Liu et al. (2010) [37]	V2	42.2/40.5	(4.5/4.7) y	30/30	E + S-ACU		Estazolam	20 days	V1	—
Liu et al. (2013) [38]	CCMD-3	(36.74 ± 9.31)/(35.66 ± 8.99)	(25.3 ± 7.6)/(25.9 ± 10.3) m	45/45	E-ACU		Diazepam	10 days	V2	—
Liu et al. (2015) [39]	CCMD-3 + ICD-10	(21 ∼ 70)/(23 ∼ 68)	(3 ∼ 32)/(4 ∼ 34) m	98/98	C-ACU		Estazolam	20 days	V4	AEs
Luo et al. (2006) [40]	CCMD-3	(39.53 ± 13.62)/(40.00 ± 13.02)	(34.78 ± 13.25)/(36.23 ± 10.54)m	32/32	C-ACU		Clonazepam	4 weeks	V2, V3	—
Luo et al. (2008) [41]	CCMD-3	(45.3 ± 4.4)/(46.2 ± 5.1)	(3.1 ± 0.1)/(3.2 ± 0.2) m	30/30	C-ACU		Estazolam	4 weeks	V1	—
Pi et al. (2018) [42]	ICD-10	(43.34 ± 12.28)/(43.45 ± 13.28)	(45.15 ± 14.72)/(48.67 ± 15.94) m	60/30	C-ACU		Estazolam	4 weeks	V5	—
Qi et al. (2008) [43]	CCMD-3	(53.73 ± 15.97)/(58.22 ± 13.82)	(14.63 ± 15.97)/(18.22 ± 13.82) m	38/38	C-ACU		Alprazolam	5 weeks	V4	AEs
Ren et al. (2017) [44]	CCMD-3	(56.8 ± 9.14)/(56.2 ± 8.69)	(52.1 ± 11.2)/(54.5 ± 12.1) m	32/32	C-ACU		Zopiclone	4 weeks	V5	AEs
Shi et al. (2016) [45]	V1	(42.51 ± 9.44)/(41.85 ± 9.97)	(19.33 ± 4.71)/(18.51 ± 5.36) m	39/36	E + S-ACU		Estazolam	4 weeks	V1	—
Song et al. (2017) [46]	V2	(46.1 ± 13.7)/(46.5 ± 13.2)	(4.3 ± 1.2)/(4.5 ± 1.1) y	39/39	W-ACU		Nitrazepam	30 days	V1	—
Wang et al. (2006) [47]	CCMD-3	16 ∼ 75	(59.16 ± 73.97)/(65.33 ± 101.33) m	90/90	C-ACU		Clonazepam	4 weeks	V2, V3	3 months
Wang et al. (2013) [30]	CCMD-3	(34 ± 5.2)/(36 ± 8.5)	4 w ∼ 10 y	25/25	E + S-ACU		Estazolam	20 days	V3	—
Wang et al. (2015) [48]	CCMD-3 + ICD-10	38.83 ± 7.04	45 d∼2 y	30/30	C-ACU		Estazolam	4 weeks	V1	—
Wang et al. (2016a) [49]	CCMD-3	(53 ± 13.43)/(53 ± 11.37)	(2.35 ± 2.02)/(2.07 ± 1.10) y	34/34	C-ACU		Estazolam	4 weeks	V2	—
Wang et al. (2016b) [50]	CCMD-3	(46.72 ± 9.16)/(47.6 ± 9.09)	(28.50 ± 10.89)/(30.13 ± 9.06) m	32/30	W-ACU		Zopiclone	2 weeks	V5	1 month
Wang et al. (2016c) [51]	CCMD-3	16 ∼ 68	3 m ∼ 10 m	44/44	E-ACU		C-ACU	30 days	V1	3 months
Wang et al. (2016d) [52]	CCMD-3	45.3 ± 2.4	Not mention	35/35	W-ACU		C-ACU	36 days	V1	—
Wang et al. (2018) [53]	CCMD-3	(46.78 ± 3.96)/(45.99 ± 4.47)	(6.03 ± 1.98)/(6.29 ± 2.14) m	39/39	C-ACU		Zolpidem	4 weeks	V1	AEs
Wu et al. (2014) [54]	ICD-10	(50.0 ± 14.3)/(50.6 ± 15.4)	(14.9 ± 8.5)/(16.9 ± 9.8) m	20/20	C-ACU		Estazolam	4 weeks	V2	AEs
Xu et al. (2014) [55]	CCMD-3	(38.6 ± 11.5)/(39.5 ± 11.6)	(5.7 ± 3.3)/(5.6 ± 3.2) m	45/30	E-ACU		Estazolam	21 days	V1	—
Xu et al. (2016) [56]	CCMD-3	20 ∼ 65	1 m ∼ 2 y	35/35	C-ACU		Estazolam	6 weeks	V3	1 months
Xuan et al. (2007) [57]	ICD-10	(47.05 ± 10.54)/(51.05 ± 13.27)	(69.75 ± 82.10)/(57.38 ± 48.29) m	24/22	C-ACU		Estazolam	30 days	V5	—
Zhang et al. (2010) [58]	CCMD-3	(37.2 ± 14.4)/(39.4 ± 13.7)	(13.1 ± 6.9)/(10.5 ± 5.1) m	28/28	S-ACU		C-ACU	30 days	V1	—
Zhang et al. (2015) [59]	V1	(42 ± 12)/(41 ± 11)	(3.50 ± 2.53)/(3.14 ± 2.55) y	38/37	C-ACU		Estazolam	20 days	V1, V3	—
Zhao et al. (2018) [60]	CCMD-3	Not mention	Not mention	30/30	E + S-ACU		C-ACU	8 weeks	V1	—
Zheng et al. (2009) [61]	CCMD-3	(59 ± 15)/(58 ± 15)	(473.76 ± 1315.12)/(333.67 ± 524.32) d	46/46	E-ACU		Estazolam	20 days	V3	—
Zhou et al. (2010) [62]	CCMD-3	(35.1 ± 12.9)/(37.4 ± 14.5)	(13.3 ± 6.7)/(10.5 ± 5.1) m	35/35	S-ACU		C-ACU	20 days	V1	—
Zhou et al. (2016) [63]	CCMD-3	(45.5 ± 12.5)/(44.7 ± 11.8)	Not mention	33/32	S-ACU		Estazolam	2 weeks	V1	—
Zhou et al. (2018) [64]	CCMD-3 + ICD-10	(49.1 ± 16.7)/(48.7 ± 15.4)	(14.0 ± 10.5)/(14.1 ± 10.4) m	30/30	C-ACU		Estazolam	4 weeks	V1	—
Zhu et al. (2015) [65]	CCMD-3	(37 ± 4.5)/(35 ± 6.7)	(22.7 ± 23.8)/(30.2 ± 24.4) m	30/30	E + S-ACU		Estazolam	21 days	V1	—

T, treatment group; C, control group; CCMD-3, Chinese Classification of Mental Disorders Third Revision; ICSD-2, International Classification of Sleep Disorders; ICD-10, International Classification of Disease Tenth Revision; CCMD-2-R, Chinese Classification of Mental Disorders Second Edition-Revision; V1, Guiding Principles for Clinical Study of New Chinese Medicines; V2, Standards for Diagnosis and Curative Eﬀect of Chinese Medical Symptom; V3, Sleep Efficiency Calculation published by WHO; V4, Guiding Principles for Clinical Study of New Chinese Medicines combined with PSQI reducing rate; V5, PSQI reducing rate; AEs, adverse events; C-ACU, conventional acupuncture; E-ACU, electroacupuncture; S-ACU, scalp acupuncture; W-ACU, warm acupuncture; E + S-ACU, electroacupuncture combined scalp acupuncture.

**Table 2:** Network meta-analysis comparisons.

W-M	**2.86** ^**∗**^ **(2.05, 3.95)**	**6.29** ^**∗**^ **(3.36, 12.67)**	**8.12** ^**∗**^ **(4.07, 16.81)**	**3.79** ^**∗**^ **(1.85, 8.16)**	**5.20** ^**∗**^ **(2.43, 11.28)**
0.35 (0.25, 0.49)	C-ACU	**2.18** ^**∗**^ **(1.10, 4.68)**	**2.86** ^**∗**^ **(1.48, 5.72)**	1.32 (0.62, 2.95)	1.81 (0.87, 4.01)
0.16 (0.08, 0.30)	0.46 (0.21, 0.91)	E-ACU	1.28 (0.51, 3.36)	0.61 (0.22, 1.59)	0.83 (0.30, 2.21)
0.12 (0.06, 0.25)	0.35 (0.17, 0.67)	0.78 (0.30, 1.97)	S-ACU	0.46 (0.17, 1.33)	0.64 (0.24, 1.67)
0.26 (0.12, 0.54)	0.76 (0.34, 1.61)	1.65 (0.63, 4.51)	2.19 (0.75, 5.94)	W-ACU	1.37 (0.47, 3.82)
0.19 (0.09, 0.41)	0.55 (0.25, 1.15)	1.20 (0.45, 3.31)	1.56 (0.60, 4.24)	0.73 (0.26, 2.12)	E + S-ACU

W-M, western medicine; C-ACU, conventional acupuncture; E-ACU, electroacupuncture; S-ACU, scalp acupuncture; W-ACU, warm acupuncture; E + S-ACU, electroacupuncture combined scalp acupuncture; ^∗^significant difference. Note. The values in the lower-left part of the table suggest the OR of the column index compared with that of the row index, and the values in the upper-right part of the table suggest the OR of the row index compared with that of the column index. OR > 1.00 of the lower-left and upper-right parts of the table indicates the high effectiveness of the intervention measures listed. Significant results are in bold.

**Table 3:** The PSRF value.

Parameter	PSRF
d.1.2	1.00
d.1.3	1.00
d.1.4	1.00
d.1.5	1.01
d.1.6	1.01
sd.d	1.02

*Note*. The parameter PSRF moves close to 1, indicating satisfactory convergence. 1, western medicine; 2, conventional acupuncture; 3, electroacupuncture; 4, scalp acupuncture; 5, warm acupuncture; 6, electroacupuncture combined scalp acupuncture (reproduced from Peng Pang et al. [66] (under the Creative Commons Attribution License/Public Domain)).

**Table 4:** Node-splitting test result.

Name	Direct effect	Indirect effect	Overall	*P* value
1, 2	1.17 (0.81, 1.52)	0.53 (−0.25, 1.29)	1.05 (0.72, 1.37)	0.13
1, 3	1.85 (1.09, 2.72)	2.05 (0.57, 3.36)	1.84 (1.21, 2.54)	0.78
1, 4	1.77 (0.69, 3.17)	2.36 (1.45, 3.31)	2.09 (1.40, 2.82)	0.48
1, 5	1.06 (0.29, 1.85)	2.83 (1.01, 5.04)	1.33 (0.61, 2.10)	0.08
1, 6	1.60 (0.68, 2.65)	1.70 (0.41, 3.03)	1.65 (0.89, 2.42)	0.92
2, 3	0.91 (−0.41, 2.33)	0.81 (−0.07, 1.68)	0.78 (0.10, 1.54)	0.88
2, 4	1.25 (0.43, 2.15)	0.69 (−0.41, 2.04)	1.05 (0.39, 1.74)	0.46
2, 5	1.75 (0.02, 4.00)	−0.03(−0.87, 0.84)	0.28 (−0.48, 1.08)	0.08
2, 6	0.71 (−0.55, 1.89)	0.51 (−0.74, 1.67)	0.60 (−0.14, 1.39)	0.82

*Note*. *P* > 0.05 means that the direct comparisons were consistent with the indirect comparisons. 1, western medicine; 2, conventional acupuncture; 3, electroacupuncture; 4, scalp acupuncture; 5, warm acupuncture; 6, electroacupuncture combined scalp acupuncture.
